# Emergence of the delta variant and risk of SARS-CoV-2 infection in secondary school students and staff: Prospective surveillance in 18 schools, England

**DOI:** 10.1016/j.eclinm.2022.101319

**Published:** 2022-02-26

**Authors:** Shamez N. Ladhani, Georgina Ireland, Frances Baawuah, Joanne Beckmann, Ifeanyichukwu O. Okike, Shazaad Ahmad, Joanna Garstang, Andrew J. Brent, Bernadette Brent, Felicity Aiano, Zahin Amin-Chowdhury, Meaghan Kall, Ray Borrow, Ezra Linley, Maria Zambon, John Poh, Lenesha Warrener, Angie Lackenby, Joanna Ellis, Gayatri Amirthalingam, Kevin E. Brown, Mary E. Ramsay

**Affiliations:** a Immunisation and Vaccine Preventable Diseases Division, UK Health Security Agency, 61 Colindale Avenue, London NW9 5EQ, UK; b Paediatric Infectious Diseases Research Group, St. George's University of London, London SW17 0RE, UK; c East London NHS Foundation Trust, 9 Alie Street, London E1 8DE, UK; d University Hospitals of Derby and Burton NHS Foundation Trust, 201 London Road, Derby DE1 2TZ, UK; e Manchester University NHS Foundation Trust, Oxford Road, Manchester M13 9WL, UK; f Birmingham Community Healthcare NHS Trust, Holt Street, Aston B7 4BN, UK; g Nuffield Department of Medicine, Oxford University Hospitals NHS Foundation Trust, Old Road, Oxford OX3 7HE, UK; h University of Oxford, Wellington Square, Oxford OX1 2JD, UK; i UK Health Security Agency, Manchester Royal Infirmary, Manchester, UK

**Keywords:** Education setting, COVID-19, SARS-CoV-2, Teenagers, Antibody testing, PCR

## Abstract

**Background:**

The role of educational settings in SARS-CoV-2 infection and transmission remains controversial. We investigated SARS-CoV-2 infection, seroprevalence, and seroconversion rates in secondary schools during the 2020/21 academic year, which included the emergence of the more transmissible alpha and delta variants, in England.

**Methods:**

The UK Health Security Agency (UKHSA) initiated prospective surveillance in 18 urban English secondary schools. Participants had nasal swabs for SARS-CoV-2 RT-PCR and blood sampling for SARS-CoV-2 nucleoprotein and spike protein antibodies at the start (Round 1: September-October 2020) and end (Round 2: December 2020) of the autumn term, when schools reopened after national lockdown was imposed in January 2021 (Round 3: March-April 2021), and end of the academic year (Round 4: May-July 2021).

**Findings:**

We enrolled 2314 participants (1277 students, 1037 staff; one participant had missing data for PCR testing). In-school testing identified 31 PCR-positive participants (20 students, 11 staff). Another 247 confirmed cases (112 students, 135 staff) were identified after linkage with national surveillance data, giving an overall positivity rate of 12.0% (278/2313; staff: 14.1%, 146/1037 *vs* students: 10.3%, 132/1276; *p* = 0.006). Trends were similar to national infection data. Nucleoprotein-antibody seroprevalence increased for students and staff between Rounds 1 and 3 but were similar between Rounds 3 and 4, when the delta variant was the dominant circulating strain. Overall, Nucleoprotein-antibody seroconversion was 18.4% (137/744) in staff and 18.8% (146/778) in students, while Spike-antibody seroconversion was higher in staff (72.8%, 525/721) than students (21.3%, 163/764) because of vaccination.

**Interpretation:**

SARS-CoV-2 infection rates in secondary schools remained low when community infection rates were low, even as the delta variant was emerging in England.

**Funding:**

This study was funded by the UK Department of Health and Social Care.

**Research in context**

**Evidence before this study**

We searched PubMed with the terms “COVID-19″ or “SARS-CoV-2″ with “school”, “education”, or “student” to identify publications relating to SARS-CoV-2 infections and COVID-19 cases in secondary schools from Jan 1, 2020 until Sept 31, 2021, focusing particularly on studies involving the more transmissible alpha and delta variants. Investigations conducted in 2020 and early 2021 found school infection rates correlated with community infection rates and were mainly due to multiple separate introductions of the virus into the educational setting, with very low secondary transmission rates within school premises when mitigations are in place. As with previously circulating SARS-CoV-2 strains, outbreaks in schools have been reported with both alpha and delta variants.

**Added value of this study**

Over the 2020/21 academic year, we found diagnosed infection rates were higher in staff than students in 18 secondary schools in England, with the majority of cases occurring between September and December 2020, as the alpha variant emerged in the UK. Serum samples showed N antibody positivity, an indicator of prior infection, increased between testing Rounds 1, 2, and 3, but changed little in Round 4. While Roche S antibody prevalence, which measures both prior infection and vaccine-induced antibodies, was higher for staff for Rounds 3 and 4 because of COVID-19 vaccinations. This corresponds with antibody seroconversion data, which found N antibody seroconversion rates were lowest for staff and students between Rounds 3 and 4.

**Implications of all the available evidence**

The reopening of secondary schools in March 2021, whilst the rest of the country remained in national lockdown, was associated with very low rates of SARS-CoV-2 infection and antibody seroconversion despite the emergence of the delta variant. The final testing round was too early to detect the infection rate increases associated with delta. On-going serosurveillance in educational settings will be important to assess the impact of vaccination on infection and transmission in educational settings.

Alt-text: Unlabelled box

## Introduction

Children have a lower risk of severe or fatal COVID-19 compared to adults.^1^ Early in the pandemic, however, schools were closed as part of national lockdown in most parts of the world, including the United Kingdom (UK), due to uncertainties of their role in transmission. As COVID-19 cases declined, some UK primary and secondary school years were partially reopened during June-July 2020 with strict infection control measures, physical distancing and smaller class sizes clustered into distinct bubbles that did not interact with other bubbles.^2^ SARS-CoV-2 infections remained low, with few outbreaks reported in educational settings, leading to the full reopening of in-person teaching in schools across the UK from September 2021.^2^^,^^3^

In England, community cases started increasing across all age-groups from August 2020 and continued to increase at the start of the first academic term of 2020/21 (Sep–Dec), with cases in children lagging behind adults until another national lockdown was imposed on 05 November 2020, although schools remained open during this period (Supplementary Figure 3).^4^ The emergence and rapid spread of the Alpha variant in the UK from mid-November 2020 led to a third national lockdown in January 2021, including school closures. Cases declined rapidly across all age-groups during January and February 2021 and remained low until mid-May 2021.^5^

From 08 March 2021, schools were reopened for three weeks whilst the rest of the country remained in lockdown and then closed for the Easter holidays. During the summer term (Mid-April to mid-July), England gradually eased out of lockdown. While cases due to the Alpha variant continued to decline rapidly from January 2021, the Delta (B.1.617.2) variant emerged in the UK in mid-March 2021 and became the most prevalent variant in England from mid-May 2021.^6^^,^^7^

Nationally, community SARS-CoV-2 infection remained low until 17 May 2021 and then started increasing rapidly, mainly in teenagers and younger adults (10–29 year olds), who were unvaccinated at the time.^5^^,^^8^ Adults were protected through COVID-19 vaccination, which began in December 2020, prioritised by age and clinical vulnerability to COVID-19, and by mid-June 2021, the vaccine was being offered all adults from 18 years of age.^9^ The combination of national lockdown, testing patterns (including twice-weekly home testing with lateral flow device (LFD) tests for secondary school students) and COVID-19 vaccination for adults changed the epidemiology of SARS-CoV-2 infection in England, such that 10–19 year-olds had the highest rates of infection after schools reopened on 08 March 2021.^10^

In order to better understand and monitor the risk of COVID-19 transmission in schools, the UK Health Security Agency (UKHSA) (formally known as Public Health England) has been conducting enhanced surveillance of COVID-19 in selected secondary schools across England since September 2021. This included PCR testing and blood sampling for SARS-CoV-2 antibodies, which captures both symptomatic and asymptomatic SARS-CoV-2 infection, in staff and students.^3^ Three earlier rounds of testing found that, by March 2021, 36% of students and 32% of staff had nucleocapsid protein (N) antibodies indicating prior infection, with an additional third of staff members having spike protein (S) antibodies through vaccination.^3^^,^^11^ Between May and July 2021, we completed a final round of testing in secondary schools when the Delta variant was prevalent across England and community cases were increasing nationally.^5^ We aim to describe SARS-CoV-2 infection and antibody seroprevalence in secondary schools between May and July 2021 and assessed trends throughout the academic year compared with national infection surveillance and testing data in England.

## Methods

The COVID-19 Surveillance in Secondary School KIDs (sKIDsPLUS) protocol is available online (https://www.gov.uk/guidance/covid-19-paediatric-surveillance),^12^ and results for the first three rounds of testing have been published.^3^^,^^11^ The study involved testing secondary school students for SARS-CoV-2 infection and antibodies at the start (Round 1: 22 September-17 October 2020) and end (Round 2: 3–17 December 2020) of the autumn term of the 2020/21 academic year, when the schools reopened in March (Round 3: 23 March-21 April 2021) and at the end of the academic year (Round 4: 20 May-14 July 2021). Participants who were isolating during Round 4 testing were sent a Tasso+ blood self-sampling device at home, which was posted back to UKHSA for antibody testing.^13^ Secondary schools were approached in areas where a paediatric investigation team could be assembled: Derbyshire, West London, East London, Greater Manchester, Hertfordshire and Birmingham. Headteachers in participating schools emailed the study information pack to staff, parents of students aged <16 years and to students aged ≥16 years. Participants 16+ years old or their parent/guardian (<16 years) provided informed consent online via SnapSurvey, and completed a short questionnaire on COVID-19 symptoms and confirmed infection prior to the sampling day or shortly afterwards. Enrolment was open for new participants between Rounds 1 and 2, although 94 participants did not participate until Round 3. A team of clinicians, nurses, phlebotomists and administrative staff attended the school on sampling days and a nasal swab and blood sample was taken for each participant. Local anaesthetic cream was offered to all students before blood sampling.

### Laboratory testing

The swabs were tested by a triplex reverse transcription PCR (RT-PCR) assay for the detection of ORF1ab and E gene regions of SARS-CoV-2 with simultaneous detection of an exogenous internal control using the Applied Biosystems Quantstudio 7-flex thermocycler (ThermoFisher Scientific, UK). The ORF1ab gene primers/probes published by the Chinese Centre for Disease Control and Prevention were combined with the E gene primers/probe published Corman et al., 2020.^14^^,^^15^ A positive RT-PCR result was reported to the participant, local investigator, head teacher and local UKHSA health protection team (HPT), typically within 48 h of the sample being taken. The participant and household members self-isolated as per national guidance. Public health risk assessment was undertaken with the school to decide additional measures, including tracing.

In Rounds 1–3, serology was performed on the Abbott Architect, using a chemiluminescent microparticle immunoglobulin G (IgG) immunoassay targeting the nucleoprotein (N) (SARS-CoV-2 IgG, Abbott Commerce Chicago, USA) with a seropositivity cut-off value of 0.8 (henceforth referred to as Abbott N assay).^16^ This assay was shown to detect SARS-CoV-2 N-antibodies as early as 7 days post-symptom onset and is, therefore, particularly useful for rapidly assessing SARS-CoV-2 antibody seroconversion (negative to positive antibodies) between testing rounds.^17^ Detectable N-antibody with the Abbott N-assay, however, falls rapidly after >3 months following SARS-CoV-2 infection, more than other commercial and in-house N-antibody assays, indicating that this is a characteristic of the assay rather than actual loss of N antibodies in most participants.^18^ Where sufficient serum was available, samples from all rounds were additionally tested for nucleoprotein and spike (S) protein antibodies on the Roche Elecsys Anti-SARS-CoV-2 N assay and Elecsys Anti-SARS-CoV-2 S assay (henceforth referred to as Roche N and Roche S assays). In Round 4, serology was performed on Roche N and S assays, due to their higher sensitivity and specificity and apparent antibody waning with the Abbott N assay, and where sufficient sample remained, samples were also tested on the Abbott N assay.^18^

### Statistical analysis

Participants were linked to national laboratory report of SARS-CoV-2 PCR and LFD tests (Second Generation Surveillance System [SGSS]) to identify diagnoses between testing rounds. A combination of unique personal National Health Service (NHS) number (obtained by linking with the Personal Demographic Service, an online national electronic database containing demographic information for all NHS-registered individuals), full name, sex, date of birth and postcode of residence was used.^19^ When calculating total diagnosed infections over the study, diagnoses identified through SGSS had to be reported between the first and last sample date for a participants school to be included.

Data were managed in R-Studio and Microsoft Access and analysed in Stata SE (version 15.1). Participants were classified as included in each round if they provided a blood sample or swab in that round. Data that did not follow a normal distribution are described as median with interquartile ranges. Categorical data are described as proportions and compared with the Chi^2^ test.

Results from the Roche N and S assay were used to present seroprevalence and estimate seroconversion rates. However, in Rounds 1 and 2, 9.7% (170/1754) and 5.9% (105/1766) persons, respectively, had insufficient remaining serum for testing on the Roche N and Roche S assays, 55% of whom were Abbott N positive. For these people, Abbott N results were used to supplement Roche N and S results. Supplementary Table 6 shows overall Abbott N antibody positivity and a comparison of Abbott N and Roche N antibody results over the study. SARS-CoV-2 infection rate and antibody seroprevalence, with 95% confidence intervals (CI), were compared between secondary school students and staff. For comparison with community seroprevalence, three-week average seroprevalence data from UKHSA and NHS Blood and Transplant (NHS BT) serosurveillance of blood donors were obtained for each school area in sKIDsPLUS. Donor sera are tested for N and S-antibodies on the Roche assays and, for this analysis, student and staff data was compared to seroprevalence in 18–30 year olds and 18–64 year old donors, respectively. In Round 4, student Roche S seropositivity do not have a comparator because vaccination rates were high in the comparator adult age-groups. Non-overlapping 95% CIs were used to assess statistical significance between student or staff rates and regional estimates.

Antibody seroconversion rates, using the Roche N and S antibody results and with 95% CIs, were calculated for participants who were tested in two sequential rounds and were negative in their first round of testing.

### Ethics statement

The protocol was approved by PHE Research Ethics and Governance Group (reference NR0228; 24 August 2020).

### Role of the funding source

This study was funded by the UK Department of Health and Social Care. The funder of the study had no role in study design, data collection, data analysis, data interpretation, or writing of the report. SNL and GI had access to the data and had final responsibility to submit for publication.

## Results

During the 2020/21 academic year (September 2020 to July 2021), 2314 participants (1277 students: 1037 staff) were enrolled and sampled at least once (Table 1). Overall 29.8% of students participated in all 4 rounds of testing, 38.4% in 3 rounds, 23.4% in 2 rounds and 8.4% in 1 round (not shown). For staff, the corresponding figures were 46.1%, 28.3%, 16.0% and 9.6%. Following Round 4, 102 students and 40 staff returned a home testing sample (Table 2). Age was significant predictors of the number of rounds students and staff participated in (*p* = 0.0015 and 0.0022 respectively), as well as school area (both *p*<0.0001) (Supplementary Table 1). Participation was higher in Rounds 1 (*n* = 1825), 2 (*n* = 1842) and 3 (*n* = 1895) than in Round 4 (*n* = 1359) (Table 2). Median time between testing rounds was 9.4 (Interquartile range [IQR]:9–11) weeks between Rounds 1 and 2, 15.9 (IQR: 15.1–15.9) weeks between Rounds 2 and 3 and 14.1 (IQR: 14–15) weeks between Rounds 3 and 4 (not shown).

**Table 1:** Characteristics of students and staff who participated in at least 1 round of sKIDs Plus.

	Total		Students		Staff	
	N	%	N	%	N	%
**Sex**						
Male	805	34.8	499	39.1	306	29.5
Female	1501	64.9	770	60.3	731	70.5
Missing	8	0.3	8	0.6	0	0.0

**Age cat**						
Lower school	692	29.9	692	54.2		
GCSEs	343	14.8	343	26.9		
A-Levels/College	242	10.5	242	19.0		
19–29	186	8.0			186	17.9
30–39	286	12.4			286	27.6
40–49	254	11.0			254	24.5
50–59	260	11.2			260	25.1
60+	51	2.2			51	4.9

**Ethnicity**						
White	1635	70.7	809	63.4	826	79.7
Black	102	4.4	58	4.5	44	4.2
Asian	361	15.6	243	19.0	118	11.4
Mixed	132	5.7	104	8.1	28	2.7
Other	64	2.8	47	3.7	17	1.6
Missing	20	0.9	16	1.3	4	0.4

**School area**						
Derbyshire	584	25.2	302	23.6	282	27.2
East London	572	24.7	316	24.7	256	24.7
Greater Manchester	181	7.8	119	9.3	62	6.0
Hertfordshire	174	7.5	106	8.3	68	6.6
West London	346	15.0	217	17.0	129	12.4
Birmingham	457	19.7	217	17.0	240	23.1

**Total**	2314		1277		1037

**Table 2:** SARS-CoV-2 PCR and antibody (Roche N and Roche S) results over 4 testing rounds in sKIDs Plus participants.

	Round 1			Round 2			Round 3			Round 4			Tasso home sampling		
	Total	Students	Staff	Total	Students	Staff	Total	Students	Staff	Total	Students	Staff	Total	Students	Staff
*PCR results*															
Negative	1817 (99.6)	943 (99.5)	874 (99.8)	1817 (99.1)	938 (98.9)	879 (99.2)	1884 (99.9)	1092 (99.8)	792 (100.0)	1339 (99.6)	689 (99.6)	650 (99.7)			
Positive	7 (0.4)	5 (0.5)	2 (0.2)	17 (0.9)	10 (1.1)	7 (0.8)	2 (0.1)	2 (0.2)	0 (0.0)	5 (0.4)	3 (0.4)	2 (0.3)			

PCR total	1824	948	876	1834	948	886	1886	1094	792	1344	692	652			

*Roche N: Antibody results*															
Negative	1467 (83.6)	742 (83.0)	725 (84.2)	1387 (78.5)	695 (77.8)	692 (79.3)	1185 (65.7)	657 (63.8)	528 (68.3)	846 (65.4)	404 (61.7)	442 (69.2)	86 (60.6)	59 (57.8)	27 (67.5)
Positive	288 (16.4)	152 (17.0)	136 (15.8)	379 (21.5)	198 (22.2)	181 (20.7)	618 (34.3)	373 (36.2)	245 (31.7)	448 (34.6)	251 (38.3)	197 (30.8)	56 (39.4)	43 (42.2)	13 (32.5)

Roche N total	1755	894	861	1766	893	873	1803	1030	773	1294	655	639	142	102	40

*Roche S: Antibody results*															
Negative	1459 (83.1)	737 (82.4)	722 (83.9)	1369 (77.5)	687 (76.9)	682 (78.1)	936 (51.9)	625 (60.7)	311 (40.2)	398 (30.8)	384 (58.6)	14 (2.2)	48 (33.8)	46 (45.1)	*
Positive	296 (16.9)	157 (17.6)	139 (16.1)	397 (22.5)	206 (23.1)	191 (21.9)	867 (48.1)	405 (39.3)	462 (59.8)	896 (69.2)	271 (41.4)	625 (97.8)	94 (66.2)	56 (54.9)	*

Roche S total	1755	894	861	1766	893	873	1803	1030	773	1294	655	639	142	102	

Round total	1825	948	877	1842	950	892	1895	1100	795	1359	700	659	142	102	

⁎ Suppressed to prevent deductive disclosure.

Of Round 4 participants, 52.6% (368/700) of students and 58.9% (388/659) of staff completed the sampling questionnaire. Since schools reopened in March 2021, most students (95.0%; 344/362) and staff (84.3%; 327/388) reported attending school every day and most students (63.7%; 230/361) and staff (76.0%; 295/388) had not missed any days off school.

### RT-PCR results

Over 4 rounds of nasal swab testing, 31 (1.3%; *N* = 2313) participants tested PCR-positive (20 students, 11 staff). Infection rates among participants varied over time and closely followed community infection rates (Table 2, Supplementary Figure 2). When linked to national testing data (PCR and LFD), an additional 247 (10.7%) participants (112 students, 135 staff) had SARS-CoV-2 diagnosed over the study period, equating to a combined total of 12.0% (278/2313) of participants testing positive. Positivity was higher in staff than students (14.1%, 146/1037; vs 10.3%, 132/1276; p-value=0.006), and most cases were between September and December 2020 (61.9%), compared to 22.7% between January and schools reopening on 08 March 2021, and 15.1% from 08 March until the final testing round (max: 14 July 2021) (Supplementary Figure 3).

Amongst participants who were not tested in Round 4, 2.5% (17/670) of students and 1.4% (6/424) of staff had confirmed SARS-CoV-2 infection reported to national surveillance in the period between 10 days prior and 6 days after the scheduled school test date (not shown). When combined with the results of those who participated in Round 4, this would equate to an overall positivity of 1.5% (20/1362) in students and 0.7% (8/1076) in staff at the final round of testing.

### Antibody prevalence

**Figure 1:**
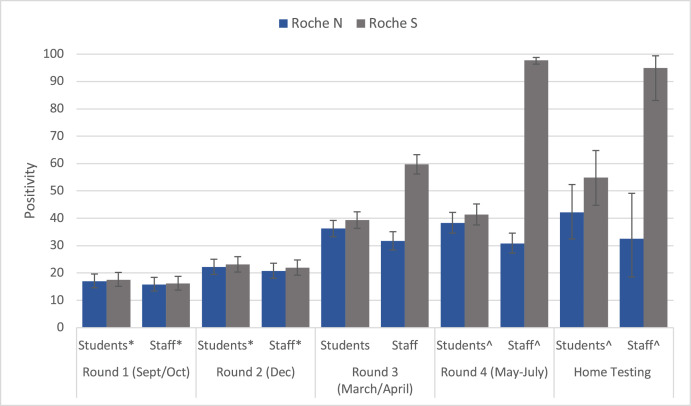
Roche N (blue) and S (grey) positivity, and 95% confidence intervals (capped lines), in student and staff sKIDs PLUS participants in round 1, 2, 3 and 4 and via home sampling.

Roche N antibody seroprevalence, indicating prior infection, increased for students and staff between Rounds 1, 2 and 3 but were similar between Rounds 3 and 4 (Figure 1, Table 2). Roche S antibody prevalence, which measures both prior infection and vaccine-induced antibodies, was similar to N antibody seroprevalence in Rounds 1 and 2 for staff and in all rounds for students, but was higher for staff for Rounds 3 and 4 because of COVID-19 vaccination, reaching >95% for staff in all school regions by Round 4. N and S antibody seroprevalence was higher, although non-significantly, in the 142 participants home-tested for SARS-CoV-2 antibodies because they missed testing in school (Table 2). In Rounds 1–4 some differences in antibody prevalence between the school areas were observed, but rates were generally similar to contemporaneous community seroprevalence estimates (Supplementary Figure 4).

### Antibody seroconversion rates

**Figure 2:**
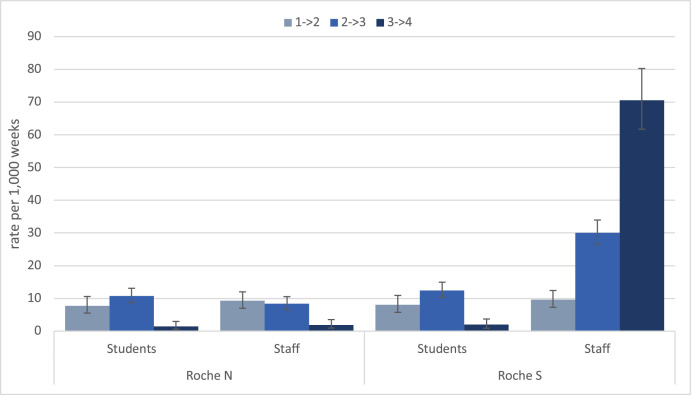
Seroconversion rates (per 1000 weeks), with 95% confidence intervals, between testing rounds in students and staff using Roche N and S antibody results in student and staff sKIDs PLUS participants. Light blue bars represent the seroconversion rate between rounds 1 and 2, medium blue bars the seroconversion rate between rounds 2 and 3 and dark blue bars the seroconversion rate between rounds 3 and 4. Capped lines denote 95% confidence intervals.

Over Rounds 1–4, 18.8% (146/778) of students seroconverted based on their N antibody results and 21.3% (163/764) based on S antibody results, compared to 18.4% (137/744) and 72.8% (525/721) of staff. N antibody seroconversion rates were lowest between Rounds 3 and 4, at 1.44 (95% CI: 0.58–2.97) students per 1000 weeks and 1.92 (95% CI: 0.92–3.53) staff per 1000 weeks (Figure 2 and Supplementary Table 5). Amongst N-antibody seroconverters who completed the questionnaire for Round 4 (52.9%; 9/17), six of nine individuals (66.7%) reported feeling unwell between Rounds 3 and 4.

## Discussion

The full reopening of in-person schooling since March 2021 was associated with very few SARS-CoV-2 infections in urban secondary schools when assessed by PCR testing and blood sampling for SARS-CoV-2 antibodies, which captures both symptomatic and asymptomatic infection 2–4 weeks after infection. Reassuringly, by mid-July 2021, 97.8% of staff had SARS-CoV-2 antibodies, consistent with the successful national COVID-19 immunisation programme for adults. Whereas ∼40% of students had acquired immunity through prior infection. Linkage with national testing data identified 12% of participants had confirmed infection during the 2020/21 academic year, with most infections occurring during the last 4 months of 2020, and infection rates closely following community infection rates at the time.

The UK situation provides unique insight into the impact of schools on community SARS-CoV-2 infection rates because schools remained open on at least two occasions when the rest of the country was in national lockdown. We previously reported that infection rates in school-aged children followed, but with a lag, adults in the community and declined following national lockdown in November 2020 despite schools remaining fully open at the time.^4^ The reopening of all schools on 08 March 2021 was associated with a small, transient increase in cases among school-aged children, driven mainly by whole-school LFD testing at the start of the term and twice-weekly home LFD testing for secondary school students. Cases did not increase in primary or secondary school students after they returned from their Easter holidays on 18 April until 17 May 2021, which coincided with the Step 3 easing of national lockdown. Despite the ready availability of testing for students, staff and their families, the proportion of undiagnosed infections, ascertained by comparing PCR/LFD diagnoses and antibody seroconversion rates, was high. Previous publications have highlighted a higher proportion of asymptomatic infection in students than staff, but twice-weekly LFD tests were recommended, which should diagnose asymptomatic cases.^3^^,^^11^^,^^20^

After 17 May 2021, the trajectory of cases in secondary-school aged students was similar to that of older teenagers and young adults who did not attend school, suggesting school re-opening was not a major factor driving the observed increase in community incidence. Rather, the easing of national lockdown likely provided more opportunities for mixing, and thereby transmission, among teenagers and young adults outside the school premises, which allowed for a more rapid spread of the virus. This would, consequently, lead to more introductions of the virus into educational settings, leading to mass educational disruption due to large numbers of students in bubbles self-isolating because of possible contact with a confirmed or suspected case.^21^

In addition to following national surveillance data, infection rate trends presented correspond with both the COVID-19 Infection Survey (CIS) (**Supplementary Figure 3a**) and School Infection Survey (SIS) over the same period, as do antibody prevalence and seroconversion data from SIS.^22^, ^23^, ^24^ SIS had a similar methodology to sKIDs Plus, with 5 testing rounds in approximately 150 schools in England, whereas CIS is a household survey that regular tests participants for COVID-19 infections.

It should be noted that face coverings and masks were mandated in classrooms for secondary school students from 08 March 2021 until 17 May 2021 because of concerns about increased transmissibility of the Alpha variant, which was the predominant circulating variant at the time. Otherwise, face coverings in secondary schools in England were only recommended for staff and students in communal areas outside the classroom if physical distancing was difficult to maintain.^25^ Whilst the contribution of face masks in preventing infections in public/community settings is well recognised, the evidence from educational settings is uncertain, especially when compared to other school mitigations (eg cancelling extra-curricular activities, restricted entry for parents/carers, outdoor instruction, daily symptom screening).^26^^,^^27^ One study found that the number of mitigations (face masks being one of them) was more important in reducing school infection rather than the individual mitigations *per se*.^27^ During the 2020/21 academic year UK schools implemented a range of infection control measures, including minimising the contact between classes, staying at home if they or a household member were symptomatic and increased hygiene and cleaning protocols.^28^ In some countries, children as young as 2 years are required to wear face coverings/masks in educational and care settings.^29^, ^30^, ^31^ There is a need for robust studies assessing the benefits and harms of face coverings/masks in young children.^32^

The large increase in childhood COVID-19 cases since June 2021, followed by a high, but stable, plateau since mid-July 2021 suggests that SARS-CoV-2 antibody seroprevalence is likely to have increased significantly in secondary school aged students since our last sampling. In the UK, adults aged ≥18 years were offered COVID-19 vaccination from mid-June 2021, and the first dose of vaccine was offered to 16–17 year-olds from 04 August 2021.^33^, ^34^, ^35^ Vaccination of 12–15 year-olds in the UK, initially with a single dose only, started on 20 September 2021.^36^ Whilst acknowledging the limited additional direct benefits of vaccination in healthy teenagers who have a very low risk of severe COVID-19, it is hoped that vaccination will help reduce further educational disruption for students.^37^ Currently, schools are fully open in England and most mitigations including bubbles removed, with only unwell children and those with confirmed COVID-19 having to self-isolate from school.

The strength of this study is the longitudinal assessment of SARS-CoV-2 infection and transmission in secondary schools. One limitation was the reduction in participation in during the final sampling visit in June/July 2021. This is the result of a large numbers of students self-isolating during this period, and. will have contributed to the apparent low infection rates on Round 4 school testing day, but linkage with national testing data found only 1.5% of students who did not take part in Round 4 had a confirmed COVID-19, indicating that most students were self-isolating because they had been a contact of a case. Reassuringly, though, the lower number of participants tested in Round 4 will not have impacted on seroprevalence and seroconversion rates between Rounds 3 and 4 because of the delay in developing an antibody response after infection and, therefore, the antibody tests performed in Round 4 would reflect infection rates at least 2–4 weeks prior to testing. Given the continuing high SARS-CoV-2 infection rates in teenagers throughout the summer months and beyond, and with COVID-19 vaccination now recommended for teenagers, it is likely that our antibody positivity rates significantly underestimate current seroprevalence. Amongst staff, participation fell because most of them were vaccinated and no longer saw study participation as an opportunity to confirm their immunity status. Additionally, student participation in enrolled schools was estimated to be 6% of the enrolled school student population over the study, although this is likely an underestimate as some schools did not actively recruit from exam-taking years. We are unable to estimate enrolment for staff as data on staff size is not available. Lastly, we only recruited schools in regions where we had paediatric investigation teams to take blood samples from large numbers of staff and students. Our schools are, therefore, not intended to be representative of all secondary schools in England and are focused on urban areas. The reopening of secondary schools since March 2021, whilst the rest of the country remained in national lockdown, was associated with very low rates of SARS-CoV-2 infection and antibody seroconversion until July 2021, despite the emergence and rapid spread of the Delta variant in England. Infection rates increased rapidly after the easing of national lockdown from 17 May 2021, however, this increase was too early to be detected in the final round of antibody sampling in July 2021. On-going serosurveillance in educational settings will be important to assess the impact of COVID-19 vaccination on infection and transmission in educational settings.

## Contributors

SNL, FB, JB, IOO, SA, JG, AJB, BB, GA, VS, KEB, and MER were responsible for conceptualisation and study design and methodology. SNL, FB, JB, IOO, SA, JG, AJB, BB, GI, FA, ZA-C, MK, RB, EL, MZ, AL, JE, LW and JP contributed to project administration (including laboratory colleagues). SNL and GI contributed to the original draft of the report, did the formal analysis and were responsible for data validation and verification. All authors contributed to reviewing and editing of the manuscripts. All authors had access to the data; SNL and GI had final responsibility to submit for publication.

## Data sharing

Applications for relevant anonymised data should be submitted to the UK Health Security Agency Office for Data Release.

## Funding

This study was funded by the UK Department of Health and Social Care.

## Declaration of interests

MR reports that The Immunisation Department also provides vaccine manufacturers (including Pfizer) with post-marketing surveillance reports about pneumococcal and meningococcal disease which the companies are required to submit to the UK Licensing authority in compliance with their Risk Management Strategy. A cost recovery charge is made for these reports. RB and EL reports performing contract research on behalf of UKHSA for GSK, Pfizer, Sanofi Pasteur, outside the submitted work. MK reports grants from Gilead Sciences Inc, outside the submitted work. MZ reports and Chair of ISIRV, Member of NERVTAG/SAGE/JCVI, Co-director NIHR HPRU, Imperial College London, Observer position on advisory boards for GSK Antivirals and Janssen RSV. JG reports Birmingham Community Healthcare NHS Trust - the employer of Dr Garstang, received expenses from Public Health England (now the UK Health Security Agency) for conducting the research in this paper. All other authors have nothing to declare.

## Supplementary materials

Image, application 1
