# Supplementary material for: Emergence of the delta variant and risk of SARS-CoV-2 infection in secondary school students and staff: Prospective surveillance in 18 schools, England
Source: eClinicalMedicine. 2022 Feb 26;45:101319. doi: 10.1016/j.eclinm.2022.101319 (PMC8882000; doi:10.1016/j.eclinm.2022.101319)
Supplement: Supplementary file 1 [file mmc1.docx]

***Supplementary Material:* Emergence of the Delta Variant and risk of SARS-CoV-2 infection in secondary school students and staff: prospective surveillance in 18 schools, England**

***Supplementary Table 1: Predictors of the number of rounds students and staff participated in, adjusted for other included variables***

|  | **Students** | |  |  | **Staff** | |  |
| --- | --- | --- | --- | --- | --- | --- | --- |
|  | Odds ratio | 95% CI | p-value |  | Odds ratio | 95% CI | p-value |
| **Sex** |  |  |  |  |  |  |  |
| Female | 1 |  |  |  | 1 |  |  |
| Male | 1.01 | 0.80-1.26 | 0.96 |  | 1.15 | 0.89-1.49 | 0.28 |
| **Age cat** |  |  |  |  |  |  |  |
| Lower school | 1 |  |  |  |  |  |  |
| GCSEs | 0.66 | 0.51-0.84 | 0.0015 |  |  |  |  |
| A-Levels/College | 0.72 | 0.53-0.97 |  |  |  |  |  |
| 19-29 |  |  |  |  | 1.4 | 0.99-1.98 |  |
| 30-39 |  |  |  |  | 1 |  | 0.0022 |
| 40-49 |  |  |  |  | 1.66 | 1.20-2.28 |  |
| 50-59 |  |  |  |  | 1.76 | 1.28-2.43 |  |
| 60+ |  |  |  |  | 2.07 | 1.16-3.68 |  |
| **Ethnicity** |  |  |  |  |  |  |  |
| White | 1 |  |  |  | 1 |  |  |
| Black | 0.98 | 0.60-1.62 |  |  | 0.73 | 0.42-1.27 |  |
| Asian | 0.72 | 0.54-0.96 | 0.19 |  | 0.75 | 0.52-1.09 | 0.46 |
| Mixed | 0.80 | 0.54-1.19 |  |  | 1.20 | 0.59-2.46 |  |
| Other | 1.10 | 0.64-1.90 |  |  | 1.03 | 0.40-2.66 |  |
| **School area** |  |  |  |  |  |  |  |
| Derbyshire | 1 |  |  |  | 1 |  |  |
| East London | 1.42 | 1.04-1.93 |  |  | 0.48 | 0.35-0.67 |  |
| Greater Manchester | 1.05 | 0.71-1.57 |  |  | 0.57 | 0.35-0.94 |  |
| Hertfordshire | 0.91 | 0.59-1.40 | <0.0001 |  | 1.78 | 1.03-3.06 | <0.0001 |
| West London | 1.96 | 1.39-2.77 |  |  | 0.90 | 0.60-1.33 |  |
| Birmingham | 2.61 | 1.84-3.69 |  |  | 1.68 | 1.20-2.35 |  |

***Supplementary Figure 2: SARS-CoV-2 positivity (overall in circles), and 95% confidence intervals (capped lines), in students (diamonds) and staff (triangles) participants over 4 rounds of secondary school testing.***

*Linked PCR and LFD testing results are not included. In round 3 no staff tested positive for SARS-CoV-2.*

***Supplementary Figure 3: a) Week of SARS-CoV-2 infection, from study PCR testing and SGSS reported diagnoses* (PCR and LFD tests), in student (dark blue) and staff (light blue) sKIDs PLUS participants over the study period (stacked bar chart) and 14-day weighted estimates of the percentage of 11/12 to 15/16 year olds testing positive for COVID-19 in England in the COVID-19 Infection Survey (circles); b) diagnosed weekly COVID-19 case rates per 100,000 population by age and timeline of lockdowns and school closures; c) variant type as a percent of all samples genotyped in England between 1 Feb and 7 August 2021***

****Diagnoses identified through SGSS had to be reported between the first and last sample date for a participants school to be included.*

*Source 3a: Office for National Statistics, 30 July 2021. COVID-19 Infection Survey: England (modelled prevalence estimates)*

*Source 3b: Public Health England. 5 August 2021. PHE Weekly National Influenza and COVID-19 Report- Week 31 report (up to week 30)*

*Source 3c: Public Health England. 20 August 2021, Variants of Concern- Technical Briefing 21.*

a)

b)

Delta variant emergence

National Lockdown & Alpha variant emergence

National Lockdown (including school closures)

Schools reopen

Tier 4 Restrictions

c)

***Supplementary Figure 4: Roche N (dark blue) and S (light blue) seroprevalence, including 95% confidence intervals (capped lines), in sKIDs Plus students and staff by school area and compared to 4-week community (regional) N (dark red) and S (pink) antibody estimates from NHSBT blood donor seroprevalence surveillance in round 1 (a), round 2 (b), round 3 (c) and round 4 (d).***

*Students are compared to 18-30 year old blood donors and staff to 18-64 year old donors. 4-week dates were; 21 Sept-18 Oct 2020 for round 1, 23 Nov-20 Dec 2020 for round 2, 15 Mar-11 Apr 2021 for round 3 and 28 Jun-25 Jul 2021 for round 4.Community S estimates are not available for round 1 and were not used in round 4 for students due to high vaccination levels in this population.*

a) Round 1

b) Round 2

c) Round 3

d) Round 4

***Supplementary Table 5: N and S antibody seroconversion in sKIDs Plus participants who were sampled in consecutive rounds on the Roche N assay (a) and Roche S assay (b).***

a)

|  |  | Sample | Seroconverters | | | |
| --- | --- | --- | --- | --- | --- | --- |
|  | |  | N | Percent | Rate per 1,000 weeks | 95% CI |
| **Round 1->2** | |  |  |  |  |  |
|  | Total | 1131 | 94 | 8.3 | 8.56 | 6.92-10.46 |
|  | Students | 523 | 39 | 9.0 | 7.76 | 5.52-10.59 |
|  | Staff | 608 | 55 | 7.5 | 9.24 | 6.97-12.01 |
| **Round 2->3** | |  |  |  |  |  |
|  | Total | 1128 | 172 | 15.2 | 9.63 | 8.25-11.17 |
|  | Students | 584 | 100 | 17.1 | 10.78 | 8.78-13.10 |
|  | Staff | 544 | 72 | 13.2 | 8.39 | 6.57-10.55 |
| **Round 3->4** | |  |  |  |  |  |
|  | Total | 734 | 17 | 2.3 | 1.69 | 0.98-2.70 |
|  | Students | 350 | 7 | 2.0 | 1.44 | 0.58-2.97 |
|  | Staff | 384 | 10 | 2.6 | 1.92 | 0.92-3.53 |

b)

|  |  | Sample | Seroconverters | | | |
| --- | --- | --- | --- | --- | --- | --- |
|  | |  | N | Percent | Rate per 1,000 weeks | 95% CI |
| **Round 1->2** | |  |  |  |  |  |
|  | Total | 1124 | 97 | 8.6 | 8.89 | 7.21-10.83 |
|  | Students | 518 | 40 | 7.7 | 8.03 | 5.74-10.92 |
|  | Staff | 606 | 57 | 9.4 | 9.60 | 7.28-12.42 |
| **Round 2->3** | |  |  |  |  |  |
|  | Total | 1111 | 368 | 33.1 | 20.93 | 18.86-23.15 |
|  | Students | 576 | 114 | 19.8 | 12.47 | 10.30-14.96 |
|  | Staff | 535 | 254 | 47.5 | 30.08 | 26.54-33.95 |
| **Round 3->4** | |  |  |  |  |  |
|  | Total | 553 | 223 | 40.3 | 29.19 | 25.53-33.22 |
|  | Students | 331 | 9 | 2.7 | 1.95 | 0.89-3.71 |
|  | Staff | 222 | 214 | 96.4 | 70.56 | 61.70-80.26 |

***Supplementary Table 6: Abbott N antibody seroprevalence in sKIDs Plus participants by round (a) and comparison of Abbott and Roche N results across sKIDs Plus (all samples included where results from both assays available, regardless of sampling round) (b).***

a)

|  | Round 1 | | |  | Round 2 | | |  | Round 3 | | |  | Round 4 | | |
| --- | --- | --- | --- | --- | --- | --- | --- | --- | --- | --- | --- | --- | --- | --- | --- |
|  | Total | Students | Staff |  | Total | Students | Staff |  | Total | Students | Staff |  | Total | Students | Staff |
| *Abbott N: Antibody results* | | | | | | | |  |  |  |  |  |  |  |  |
| Negative | 1,561 (89.0) | 779 (87.2) | 782 (90.8) |  | 1,532 (86.7) | 776 (86.9) | 756 (86.6) |  | 1,423 (79.1) | 802 (77.9) | 621 (80.5) |  | 913 (84.5) | 438 (83.1) | 475 (85.9) |
| Positive | 193 (11.0) | 114 (12.8) | 79 (9.2) |  | 234 (13.3) | 117 (13.1) | 117 (13.4) |  | 377 (20.9) | 227 (22.1) | 150 (19.5) |  | 167 (15.5) | 89 (16.9) | 78 (14.1) |
| PCR total | 1,754 | 893 | 861 |  | 1,766 | 893 | 873 |  | 1,800 | 1029 | 771 |  | 1,080 | 527 | 553 |

b)

| Abbott Results | Roche N Result | |  | Total |
| --- | --- | --- | --- | --- |
|  | Negative | Positive |  |  |
| Negative | 4,708 (99.1%) | 721 (43.7%) |  | 5,429 |
| Positive | 42 (0.9%) | 929 (56.3%) |  | 971 |
| Total | 4,750 | 1,650 |  | 6,400 |

*Percent of column totals*
